# Placenta Prævia

**Published:** 1882-10-20

**Authors:** J. D. Strawbridge

**Affiliations:** Danville, Penn.


### PLACENTA PRÆVIA.

By J. D. STRAWBRIDGE, M. D., OF DANVILLE, PENN.

Early in the summer of 1851 a woman ran crying from a house which I at that moment happened to be passing, and I turned to see what was the matter, when, recognizing me, she exclaimed: "Oh! Doctor, come up quick, Mrs. L. is dying." Following up stairs as rapidly as possible, I found the patient lying on her left side, her back toward me, near the edge of the bed, through which blood flowed in a stream to the floor. I inquired, "Is she in labor?" and the reply, "I guess so," was scarcely given, when a torrent of blood poured over the side of the bed, flooding for some distance the uncarpeted floor. A low, stifled moan, gasping breathing, ineffectual efforts at vomiting, the blanched face, and almost complete unconsciousness, showed clearly that there was no time to lose. I instantly threw off my coat, bared my right arm, and introducing my hand into the vagina, through a relaxed and easily dilated vulva, passed my finger rapidly around inside the os, which I found relaxed and dilated to nearly two inches in diameter, and through which a mass of placenta presented. I searched for some point of detachment by which to reach the membranes, but found the placenta firmly adherent all around to within little more than half an inch of the edge of the os. The fœtus could be felt through the placenta, but moving so freely in the abundant waters as to evade the touch, and prevent the presentation being ascertained. Finding a slight break in the presenting placental mass, directly across its centre, without hesitation or a moment's delay, I thrust my hand in this opening, through the placenta into the uterus, giving exit to a large flow of waters; grasping the vertex, which came at once into my hand, I gave it a turn to the left, then placing my finger in front of the right shoulder, while the waters were still discharging, rotated the body on its axis until the feet were brought nearly to the front, where they were easily reached, the child turned and delivered, within ten minutes from the time I entered the room.

The secundines, inverted through the torn placenta, followed immediately after the head. The child, which was in the beginning of its seventh month, showed no signs of life, and no effort was made to resuscitate it. As soon as it could be laid to one side, I administered to the mother (who had partially recovered consciousness), a teaspoonful of tincture of camphor in water (the only stimulant at hand), and a few minutes later 30 grains of powdered ergot in infusion. There was no further hemorrhage, and the patient, although in a state of extreme exhaustion, recovered rapidly, without a bad symptom, and within a month was able to look after the affairs of the household. The placenta proved to have been placed centrally over the internal os, and was torn through, close to the insertion of the cord.

As soon after as my patient could be safely left I called upon Dr. Wm. H. Magill for advice in this case, and learned that he had had an almost similar experience with the same patient about one year before. Mrs. L. being then in her first pregnancy, and, as she supposed, in her sixth month, was suddenly taken with a large flow of blood while standing at work; she was put to bed as soon as possible, and her physician, Dr. W. R. Gearhart, called; finding no signs of labor, the vagina was tamponed and the Doctor left to attend to other calls; returning a few hours later, he found the patient faint and weak, and, notwithstanding the presence of the tampons, the hemorrhage was becoming dangerous. Alarmed at this condition, he sent in haste for Dr. Magill, who at once removed the tampons; finding the os sufficiently dilated, he detached the placenta at the side, ruptured the membranes with his fingers, and rapidly effected version and delivery by the feet; although no delay occurred in the delivery, and but little force was required, the child showed no signs of life.

A little less than one year from my first attendance I was again called in haste to Mrs. L., and found her in bed, having been taken suddenly a few minutes before, with profuse hemorrhage. She was then near the end of her sixth month of pregnancy, and, certain that I had again a placenta prævia to deal with, I had in my pocket a package containing three drachms of powdered ergot, which I handed to one of the attendants, with directions to put it in half a teacupful of boiling water, and return with it immediately. While preparing for an examination, a sudden gush of blood, which filled the bed and clothing about the patient, warned me that there was no time for delay, or the catastrophe so narrowly averted on the former occasion might be realized on this. I found complete dilatation of the soft parts, some small coagula still in the vagina, and blood flowing in a continuous stream from the partially dilated os, through which a mass of placenta presented; a partial detachment at one side, where a forming bag of waters could be felt, enabled me to rupture the membranes at once and pass my hand up into the uterus, as the waters flowed rapidly away, and the head began to come down. I found the pressure of my hand had almost completely arrested the hemorrhage. The attendant was directed to administer one-third of the infusion of ergot, and I determined to await results. At the end of fifteen minutes a second portion was given, and in about fifteen minutes more uterine contractions began, and soon the head was pressing down firmly into my hand, which I began slowly to withdraw, taking care not to relax pressure on the placenta until it was compressed firmly by the head. The labor progressed rapidly, and within an hour from my arrival a dead child was delivered. The patient was but little more exhausted than in an ordinary labor, and was able to be up on the tenth day.

In December of the same year I learned from Mrs. L. that she was again pregnant, and dreaded a recurrence of her former trouble. I charged her to go to bed and send at once for a physician on the appearance of the slightest hemorrhage. Accordingly, in the latter part of January, 1853, I was called, and found her in bed, having had a slight show, with some fugitive pains about the back and abdomen. A digital examination showed no signs of labor. She believed herself then at the middle of her seventh month. I enjoined rest in bed, cold drinks, and prescribed a few doses of acetate of lead and opium; in a few hours the hemorrhage ceased, and the next day, in spite of my injunction, she was again about the house. These attacks returned at intervals of about a week, increasing in quantity and duration of flow with each return, for which, after the first attack, I prescribed, to be alternated with the acetate of lead and opium, five grains of powdered ergot every four hours. About the middle of March I was called in haste, and found her in bed, with regular labor pains, accompanied with free hemorrhage; the os dilated to the size of a silver dollar, through which the edge of the placenta protruded. Pressing this to one side, I ruptured the membranes; the pains increased rapidly, and soon the vertex presented. With the pressure of the head on the placenta the hemorrhage soon ceased. I felt that I could trust the natural powers for a satisfactory result. After an easy labor of about two hours, my patient was the mother of a fine, healthy boy, now a successful business man in the State of Ohio and the father of several healthy children. The family were preparing to leave Danville, and soon after went to their new home in Ohio.

While visiting a patient a few weeks since, I was introduced to Mrs. L., a fine, matronly-looking widow lady, of about fifty years; to my great surprise I found she was my former patient, whom I had not seen or even heard of for over twenty-seven years. At this meeting all the circumstances here related were recalled and discussed. I learned that after leaving Danville she had given birth to several children, without any complication whatever, but none of whom lived to grow up. She promised to see me again and furnish me more accurate data of her case, my own records having been lost while absent from home during the war; but professional duties called me from home, and Mrs. L. left Danville without seeing me again. This, with but one exception, where the condition was but partial, is the only patient in my own practice in whom I have met with "placenta prævia," although, between my last attendance on Mrs. L., in the spring of 1853 and the fall of 1880, when I temporarily retired from practice, I attended in more than 1,700 cases of labor.—*Med. and Surg. Rep.*

---

**An Emergency Case.**—Professor: "What would you do, sir, if you were called to see a man who had hung himself?" Student: "I would cut him down." Professor: "Then what would you do?" Student: "I would cut him up."—*Punch.*

